# Factors associated with acquisition of carbapenem-resistant Enterobacteriaceae^1^

**DOI:** 10.1590/1518-8345.1751.2935

**Published:** 2017-10-05

**Authors:** Lilian Silva Lavagnoli, Bil Randerson Bassetti, Thais Dias Lemos Kaiser, Kátia Maria Kutz, Crispim Cerutti

**Affiliations:** 2 MSc, Microbiologist, Laboratório de Microbiologia Médica, Secretaria de Saúde, Vitória, ES, Brazil.; 3 Physician, Hospital Estadual Central, Vitória, ES, Brazil. Physician, Hospital Santa Casa de Misericóridia de Vitória, Vitória, ES, Brazil.; 4 Specialist in Applied Microbiology, Microbiologist, Hospital Santa Casa de Misericóridia de Vitória, Vitória, ES, Brazil.; 5 PhD, Associate Professor, Universidade Federal do Espírito Santo, Vitória, ES, Brazil.

**Keywords:** Enterobacteriaceae, Drug Resistance, Microbial, Risk Factors, Epidemiology, Dissemination of Resistance, Hospital Environment

## Abstract

**Objective:**

to identify possible risk factors for acquisition of Enterobacterial strains with a marker for resistance to carbapenems.

**Methods:**

exploratory case-control study performed in hospital settings. The study sample consisted of patients with biological specimens that tested positive for carbapenem-resistant Enterobacteriaceae (cases), with the disk diffusion test and Etest, and controls with biological samples testing negative for carbapenem-resistant Enterobacteriaceae. In all, 65 patients were included: 13 (20%) cases and 52 (80%) controls.

**Results:**

the microorganisms isolated were Serratia marcescens (6), Klebsiella pneumoniae (4), and Enterobacter cloacae (3). Univariate analysis revealed that length of hospitalization prior to sample collection (p=0.002) and having a surgical procedure (p=0.006) were statistically significant. In the multivariable logistic regression model, both were still significant, with odds ratios of 0.93 (p = 0.009; 95% CI: 0.89 to 0.98) for length of hospitalization prior to sample collection, and 9.28 (p = 0.05; 95% CI: 1.01 to 85.14) for having a surgical procedure.

**Conclusion:**

shorter hospitalization times and increased surveillance of patients undergoing surgery could play a decisive role in reducing the spread of carbapenem-resistant microorganisms in hospital settings.

## Introduction 

Members of the Enterobacteriaceae family are Gram-negative microorganisms found in nature, and isolated from biological material, that colonize the gastrointestinal tract of humans as part of the normal microbiota of this organ system, making it a potential reservoir for these pathogens. Carbapenem-resistant Enterobacteriaceae (CRE) have emerged as an important cause of nosocomial infections around the world, and are characterized by rapid, progressive dissemination^1^. They are currently an important worldwide public-health problem, as infections due to CRE result in a high mortality rate, with limited therapeutic options^2^ ^-^ ^3^.

Production of β-lactamase enzymes that can hydrolize carbapenems (carbapenemases) is one of the main mechanisms of resistance in Enterobacteriaceae. According to the existing classification, carbapenemases belong to molecular class A (Klebsiella pneumoniae carbapenemase - KPC), B (metallobetalactamases, of which the primary ones are types VIM, IMP and NDM), and D (the most important being type OXA-48)^4^. The KPC is one of the most epidemiologically important types, because of its worldwide dissemination^5^.

Carbapenemases can be transferred between different strains of bacteria, usually by small circular DNA (deoxyribonucleic acid) molecules known as plasmids^4^, which can replicate independently from chromosomal DNA, and allow genetic material to be exchanged between different genera and species of Enterobacteriaceae^6^. This horizontal transfer of genes can involve multiple pathogens, and become widespread in a hospital setting.

The molecular epidemiology of carbapenem-resistant bacteria has been extensively investigated. However, most of the available information comes from studies that investigated specific bacteria^7^ ^-^ ^10^ or specific types of infection^11^ ^-^ ^12^. The risk factors associated with the transmission of resistant pathogens cannot be fully understood when investigations are limited to specific bacteria, because plasmids with resistant traits can be transferred between bacteria of different species. Investigations of infection and colonization by CRE should therefore be more general, and not specify the genus of the bacteria or the patient’s clinical condition^1^ ^,^ ^13^ ^-^ ^16^. Hence, there is a need for a study with a more comprehensive case definition, to provide a better understanding of the risk factors for infection by these microorganisms, so that effective prevention and control measures can be implemented. 

The aim of the present exploratory case-control study was to identify possible risk factors for acquisition of Enterobacterial strains with a marker for carbapenem resistance. 

## Method

### Study design

This case-control study involved patients seen at one public and one not-for-profit hospital (a non-governmental non-profit facility serving the public health system), each with 300 inpatient beds, in Vitória, ES, Brazil. The hospital infection rates were similar for both hospitals during the study period. The target population was composed of all individuals hospitalized in the two institutions who had suspected nosocomial infection. The sample was composed of individuals with a confirmed presence of CRE by the Medical Microbiology Laboratory of the Central Laboratory Complex (LACEN/ES), between January 1, 2013, and July 31, 2014 (denominated cases). For each case, four randomly selected individuals with laboratory tests negative for CRE or any other organism, who were in the same unit at the same time as the case (± 20 days), composed the matched controls. Individuals whose records contained less than 50% of the information needed were excluded from the study. Controls whose records had insufficient information were replaced by other randomly selected controls. Approval for the study was granted by the Committee for Ethics in Research at the Center for Health Sciences, Federal University of Espírito Santo (ref. no. 908.781).

### Microbiological procedures

Cultures sent to LACEN/ES were first tested biochemically to investigate bacterial metabolism (Himedia, Mumbai, India) in order to identify the genus/species of the bacteria isolated. The biochemical tests included glucose, sucrose and lactose fermentation; CO_2_ production; motility; indole production; urea hydrolysis; lysine, arginine and ornithine decarboxylase activity; citrate and malonate utilization; phenylalanine deaminase activity; and H_2_S production^17^.

Once the bacteria had been identified, samples were tested for antimicrobial susceptibility by disk diffusion on Mueller-Hinton agar (Oxoid, Hampshire, United Kingdom) and Etest (Biomerieux, Marcy-l’Étoile, France) to confirm the carbapenem-resistance profile (resistance to ertapenem, imipenem or meropenem), in accordance with standards from the Clinical and Laboratory Standards Institute, ^18^ ^-^ ^19^ and modifications in the Brazilian Health Surveillance Agency (Agência Nacional de Vigilância Sanitária - ANVISA) technical notes (http://www.anvisa.gov.br). The samples were also screened using the modified Hodge test to detect carbapenemases^18^ ^-^ ^19^, the Etest (Biomerieux, Marcy-l’Étoile, France) to detect extended spectrum betalactamase (ESBL)-producing and metallobetalactamase (MBL)-producing bacteria and disk diffusion on Mueller-Hinton agar (Oxoid, Hampshire, United Kingdom), to detect AmpC betalactamase-producing bacteria.

Strains identified as CRE (i.e., strains resistant to ertapenem, imipenem or meropenem) were sent to the Nosocomial Infection Research Laboratory at the Oswaldo Cruz Foundation in Rio de Janeiro (LAPIH/FIOCRUZ) in tubes with a nutrient agar slant (Himedia, Mumbai, India), for identification of resistant genes by an in-house Polymerase Chain Reaction (PCR) technique to detect the *bla*_KPC_ gene. The Collection of Bacterial Cultures of Hospital (CCBH) Origin 4640 strain (*K. pneumoniae* ST437 - KPC-2) was used as the positive control, and the American Type Culture Collection (ATCC) 700603 strain (*K. pneumoniae* ESBL positive) as the negative control. The primers used were KPC-A (5´-CTGTCTTGTCTCTCATGGCC-3´) and KPC-B (5´-CCTCGCTGTGCTTGTCATCC- 3´)^20^.

After PCR amplification, the products were processed on 1.5% agarose gel, and electrophoresis was performed in a Tris, Borato and EDTA (TBE) 0.4X buffer at room temperature, with a voltage between 80 and 120 V for approximately 30 minutes. To visualize the amplified products after the run, the gel was stained with ethidium bromide to a final concentration of 0.5 µg/mL for 17 minutes, and destained in water for 15 minutes. The gel was then visualized under ultraviolet (UV) light and photographed using a Polaroid Gel Doc photodocumentation system. The method used followed the LAPIH/FIOCRUZ protocol.

### Variables 

The following items were investigated as possible risk factors: gender; age; hospitalization during the previous 90 days; hospitalization in an intensive care unit; use of a catheter or other invasive device; surgery during the current hospitalization; underlying comorbidities; and antimicrobial agents used during the current hospitalization. Unless otherwise stated, the events and periods considered in the analysis occurred before the biological samples were collected.

### Sampling

To increase the power of the study, four controls hospitalized in the same unit during the same period as the cases were randomly selected and assigned to each case, giving a total of 13 cases and 52 controls enrolled in the study.

### Statistical analysis

Numeric variables were summarized using measures of central tendency and variability. Medians and interquartile ranges were used, as the data did not have a symmetric distribution. Categorical variables were summarized by their absolute frequencies and their proportions in each category.

To investigate the association between the different variables in the data collection instrument and the outcome in question (the presence or absence of CRE), the variables were compared using a univariable logistic regression model. Variables for which the association with the outcome had a p-value of less than 0.2 were included in the multivariable model.

The multivariate analysis was performed using multivariable conditional logistic regression. Effect measures were calculated using the odds ratio, and respective 95% confidence interval. Goodness-of-fit was assessed by the Hosmer-Lemeshow test^21^. The data were analyzed in Statistical Package for the Social Sciences (SPSS), version 17.

## Results

**Figure 1:**
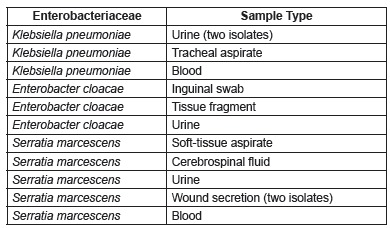
Species of Enterobacteriaceae isolated from the 13 cases and the source of each isolate. Vitória, ES, Brazil, 2015

Figure 1 shows the Enterobacteriaceae species isolated in the 13 cases. Table 1 shows demographic and clinical characteristics of cases and controls. Only three of the records selected had to be replaced because of incomplete data. Univariate analysis of the variables, analyzed for their association with the outcome represented by colonization or infection by CRE, showed that length of hospitalization prior to sample collection (p=0.002) and having a surgical procedure (p=0.006) were statistically significant (Table 1). All the variables that had a p-value of less than 0.2 in the initial stage were included in the logistic regression model (Table 2). 

**Table 1:** Univariate analysis of variables potentially associated with colonization and infection by carbapenem-resistant Enterobacteriaceae. Vitória, ES, Brazil, 2015

**Characteristics**		**Cases (N=13)**	**Controls (N=52)**	Odds Ratio [95% CI*] (p-value) ^**†**^
**Gender**				**0.63 [0.18-2.13] (0.46)**
	**Male**	**6 (46.2%)**	**30 (57.7%)**	
	**Female**	**7 (53.8%)**	**22 (42.3%)**	
**Age**				**0.99 [0.97-1.02] (0.74)**
	**Median**	**54.0 years**	**65.5 years**	
	**Interquartile range**	**46.0 to 79.0 years**	**46.5 to 78.3 years**	
**Length of hospitalization prior to sample collection**				**0.92 [0.88-0.97] (0.002)**
	**Median**	**34 days**	**12 days**	
	**Interquartile range**	**27 to 93.5 days**	**5.2 to 21 days**	
**Surgical procedure**				**9.55 [1.91-47.74] (0.006)**
	**Yes**	**11 (84.6%)**	**19 (36.5%)**	
	**No**	**2 (15.4%)**	**33 (63.5%)**	
**Previous hospitalization (90 days)**				**0.86 [0.25-2.90] (0.80)**
	**Yes**	**6 (46.2%)**	**26 (50%)**	
	**No**	**7 (53.8%)**	**26 (50%)**	
**Hospitalization in intensive care unit**				**4.36 [0.88-21.67] (0.07)**
	**Yes**	**11 (84.6%)**	**29 (55.8%)**	
	**No**	**2 (15.4%)**	**23 (44.2%)**	
**Use of catheters and/or invasive devices**				**3.09 [0.76-12.52] (0.12)**
	**Yes**	**10 (76.9%)**	**27 (51.9%)**	
	**No**	**3 (23.1%)**	**25 (48.1%)**	
**Comorbidities** ^**‡**^				**1.65 [0.32-8.50] (0.55)**
	**Yes**	**11 (84.6%)**	**40 (76.9%)**	
	**No**	**2 (15.4%)**	**12 (23.1%)**	
**Use of antibiotics**				**4.00 [0.47-33.81] (0.20)**
	**Yes**	**12 (92.3%)**	**39 (75%)**	
	**No**	**1 (7.7%)**	**13 (25%)**	
**Total**		**13**	**52**	**-**

*CI: Confidence Interval

†Univariable logistic regression model.

‡ Most frequently detected comorbidities: arterial hypertension (35.4%), diabetes mellitus (24.6%), heart disease (13.8%), HIV infection (12.3%), cancer (7.7%) and stroke (4.6%).

**Table 2:** Multivariate analysis of risk factors for colonization and infection by carbapenem-resistant Enterobacteriaceae. Vitória, ES, Brazil, 2015

**Characteristics**	Odds Ratio [95% CI *] (p-value)
**Length of hospitalization prior to sample collection**	**0.93 [0.89 - 0.98] (p=0.009)**
**Hospitalization in intensive care unit**	**1.69 [0.09 - 31.62] (p=0.72)**
**Use of catheter and/or invasive device**	**1.68 [0.13 - 22.19] (p=0.69)**
**Surgical procedure**	**9.28 [1.01 - 85.14] (p=0.05)**

*Confidence interval.

 Both variables remained significant in the multivariable logistic regression. Length of hospitalization prior to sample collection had an odds ratio of 0.93 (p = 0.009; 95% CI: 0.89 to 0.98), and surgical procedure had an odds ratio of 9.28 (p = 0.05; CI 95% = 1.01 to 85.14). In other words, there was a 6.6% reduction per day of hospitalization avoided in the risk of CRE being isolated. The measure of effect for surgical procedure revealed a nine times greater possible risk of having samples positive for CRE for patients who underwent these procedures, with a very large confidence interval. This large interval indicates that the estimates from the logistic model are probably unstable, due to the small number of non-surgical cases (only two out of thirteen).

Of the 13 CRE isolates tested for resistance genes using PCR, nine (69.2%) were positive for the *bla*_KPC_ gene: four isolates of *K. pneumoniae*, three of *Enterobacter cloacae* and two of *Serratia marcescens.*

## Discussion

Our findings show that there was a statistically significant association between isolation of CRE and either length of hospitalization prior to sample collection, or surgical procedure. This association remained significant in the multivariable logistic regression model. Patients who were positive for CRE had a 34-day median length of hospitalization prior to sample collection. This agrees with studies in which the length of hospitalization prior to sample collection is reported to vary from two to four weeks^22^ ^-^ ^23^. This finding can be used to characterize CRE infection as a late-onset nosocomial complication. 

Having surgery was a risk factor for acquiring CRE. This finding is in agreement with a previous study that described surgery as being more common in patients with CRE infection, and corroborates the finding that medical procedures play a significant role in increased susceptibility of hospitalized patients to certain infections^24^.

Some of the cases in this study were infected with isolates of CRE that carried the *bla*_KPC_ gene, and others were infected with isolates that did not. The decision to include both types of case was taken to allow a broader case definition, to help identify associations between different factors and the presence of multiresistant bacteria, and thereby prevent their dissemination, which is of prime importance in nosocomial epidemiology.

However, the study had several limitations. Because it was retrospective, some important information was missing from the hospital database, and could not therefore be used in the analysis. This may have introduced selection or information bias. The variability of the quantitative data reflects the limited precision resulting from the small number of observations, and indicates limited statistical power, which in turn can mean that valid associations may not have been identified. The large intervals also reveal some instability of the estimates of the coefficients in the model, preventing precise estimation of the strength of the associations. Control patients were randomly selected without a strictly established criterion for matching, which is suitable for an exploratory case-control study, but can lead to confounding. Nevertheless, confounding was probably reduced because only controls that had been in the same hospital unit at the same time as the cases were selected. Furthermore, because of its retrospective nature, as the data were obtained from medical records and not directly from the patients, the study did not provide information about possible previous colonization of control patients by CRE.

## Conclusion

This study investigated risk factors associated with CRE colonization or infection in different inpatient units in two hospitals. The findings show that, independent of the Enterobacteriacea isolated, the type of infection, or the inpatient unit, length of hospitalization and having a surgical procedure increase the probability of acquiring CRE. 

These results highlight the importance of taking effective preventive measures to avoid the spread of CRE in hospital settings. This is particularly important for health professionals, as they have free access to the inpatient units in a hospital and are in direct contact with hospitalized patients. Therefore, correct cleaning and disinfection procedures complying with regulatory agency guidelines should be followed.

The high potential for spreading CRE in a hospital setting makes effective preventive measures essential. Knowledge of the risk factors associated with acquisition of CRE, and implementation of preventive measures, such as decreasing hospitalization times and increasing surveillance of surgical patients, could play a decisive role in reducing the spread of these microorganisms in hospital settings.
